# Macular vessel density, branching complexity and foveal avascular zone size in normal tension glaucoma

**DOI:** 10.1038/s41598-020-80080-z

**Published:** 2021-01-13

**Authors:** Kelvin K. W. Cheng, Beatrice L. Tan, Lyndsay Brown, Calum Gray, Eleonora Bianchi, Baljean Dhillon, Tom MacGillivray, Andrew J. Tatham

**Affiliations:** 1 Princess Alexandra Eye Pavilion, NHS Lothian, 45 Chalmers Street, Edinburgh, EH3 9HA UK; 2 Centre for Clinical Brain Sciences, University of Edinburgh, Edinburgh, UK

**Keywords:** Image processing, Software, Diagnostic markers

## Abstract

The aim of this study was to investigate the relationship between glaucoma severity and perifoveal vessel density (pfVD), branching complexity, and foveal avascular zone (FAZ) size in normal tension glaucoma (NTG). 31 patients with NTG washed out of glaucoma medications were subjected to tests including; intraocular pressure measurement; standard automated perimetry; optical coherence tomography (OCT) measurement of macular ganglion cell complex (mGCC), inner macular thickness (IMT) and circumpapillary retinal nerve fibre layer (cpRNFL); and OCT angiography measurement of pfVD, FAZ perimeter and multispectral fractal dimensions (MSFD). Eyes with more severe glaucoma had significantly thinner mGCC and cpRNFL and lower pfVD. MD decreased by 0.4 dB (95% CI 0.1 to 0.6 dB, *P* = 0.007) for every 1% decrease in pfVD. Lower MSFD was observed in eyes with lower pfVD and in patients with systemic hypertension. Multivariable analysis, accounting for age and OCTA quality, found lower pfVD remained significantly associated with thinner IMT, thinner mGCC and worse MD but not with MSFD. pfVD was reduced in NTG and was diminished in eyes with worse MD. Macular vessel branching complexity was not related to severity of visual field loss but was lower in patients with systemic hypertension.

## Introduction

Glaucoma is a heterogenous group of optic neuropathies causing characteristic changes to the optic nerve head and retinal nerve fibre layer (RNFL), with associated loss of visual field^1^. Though glaucoma is normally associated with raised intraocular pressure (IOP), a significant proportion of patients develop glaucoma with statistically normal levels of IOP, referred to as normal tension glaucoma (NTG)^2–5^.

The pathogenesis of NTG is incompletely understood, but reduced ocular blood flow may contribute to its onset and progression^6^. An association with migraine, Raynaud’s phenomenon, and primary vascular dysregulation syndrome, in addition to a reported increased propensity for optic disc hemorrhages, suggests that vascular dysfunction is important^7^.

Optical coherence tomography angiography (OCTA) provides a non-invasive method of assessing retinal vasculature as a proxy of microvascular health and recent studies have shown a reduction in vessel density within the optic nerve head, peripapillary region, and macula (pfVD) in patients with glaucoma^8–10^. Whether changes in pfVD measured using OCTA are a result of, or contribute to, glaucomatous neural losses remains uncertain; however, impaired perfusion of the optic nerve head and retinal ganglion cell bodies has been suggested to play an important role in glaucoma progression, and this may be of particular importance in patients who develop glaucoma at statistically normal levels of IOP^8,11,12^.

OCTA therefore offers the potential to provide novel biomarkers of glaucoma, that may supplement more commonly used measures obtained from optical coherence tomography (OCT), such as circumpapillary retinal nerve fibre layer (cpRNFL) and macular ganglion cell complex (mGCC) thickness. Novel biomarkers may improve ability to detect glaucoma and glaucoma progression, improve ability to determine treatment effect, and better understand glaucoma pathogenesis.

Other potential markers of vascular integrity, not currently quantified using commercially available imaging device software, include measures of the complexity of vessel branching pattern and of the size and shape of the foveal avascular zone (FAZ). Fractal analysis is a technique for quantifying complex geometric patterns in biological structures including the retinal vascular tree, with multispectral fractal dimensions (MSFD) an index used to quantify the complexity of vessel branching. MSFD has proved its usefulness as a novel parameter in several investigations into the use of retinal imaging for revealing signs of disease elsewhere in the human body including cardiology and endocrinology^13–16^. Fractal dimensions have also been studied in neurodegenerative diseases, with multiple studies reporting differences in fractal dimensions in Alzheimer’s disease, cognitive impairment and strokes^17–21^.

Vessel Assessment and Measurement Platform for Images of the Retina (VAMPIRE) is a software application developed to allow efficient, semi-automatic quantification of retinal vessel characteristics based on fundus camera images^22^. This has been used to demonstrate retinal microvascular abnormalities in several diseases including hypertension and lacunar stroke^23,24^. Although VAMPIRE was originally used to analyze fundus photos, it has been adapted to examine OCTA images, specifically to measure MSFD and the FAZ.

Fractal dimensions of the blood vessels around the optic nerve head has been shown to be reduced in patients with advanced primary open angle glaucoma and associated with ocular hypertension when assessed using fundus photographs^25,26^. To the best of our knowledge, this is the first study examining MSFD using macular OCTA images in NTG. The aim of the current study was to further explore potential vascular biomarkers for glaucoma by examining pfVD, MSFD and FAZ size in a cohort of patients with NTG and examining the relationship between these metrics and conventional measures of glaucoma severity from standard automated perimetry (SAP) and OCT. This is the first study to apply the VAMPIRE analysis software for OCTA to patients with glaucoma.

## Methods

A prospective observational study was conducted involving patients with NTG attending Princess Alexandra Eye Pavilion, Edinburgh, UK. All participants had no recorded history of ever having had an IOP > 21 mmHg; and all provided written informed consent prior to study inclusion. Methods were prospectively approved by the South East Scotland Research Ethics Committee and adhered to the principles of the Declaration of Helsinki.

Participants were required to be willing to withhold IOP lowering treatments during a washout period on the understanding that in the opinion of the investigator they could do so without significant risk. Those who had undergone any form of previous glaucoma surgery or glaucoma laser treatment were excluded; as were potential participants who had undergone previous corneal surgery, including laser refractive surgery, or who had any concomitant corneal disease. We also excluded patients with diabetes mellitus due to the potential effect on ocular blood flow.

A comprehensive ophthalmological examination was performed including slit lamp examination, gonioscopy, dilated fundoscopy, pachymetry (Accutome PachPen, Keeler Ltd, Windsor, UK), standard automated perimetry (SAP, 24-2 Swedish Interactive Threshold Algorithm (SITA) Fast, Humphrey Field Analyzer (Carl Zeiss Meditec, Cambridge, UK). Glaucoma was defined by the presence of characteristic changes to the optic nerve head or retinal nerve fibre layer (RNFL), in addition to the presence of a glaucomatous visual field defect on SAP. Details of past medical history and current medications were recorded, with particular attention to whether there was a history of systemic hypertension. Family history of glaucoma in a first degree relative was also recorded.

Following the screening procedure, eligible patients were instructed to stop IOP-lowering medications for a washout period of up to 42 days depending on medication. Washout was for a minimum of 28 days for prostaglandin analogues and beta-blockers, 14 days for alpha-agonists, and 4 days for carbonic anhydrase inhibitors. Patients in whom washout was not deemed safe were excluded from the study.

Following washout, patients attended for a further examination. IOP off medication was assessed using the Ocular Response Analyzer (ORA G3, Reichert, Buffalo, NY, USA) to measure Goldmann correlated IOP (IOPg), corneal compensated IOP (IOPcc) and corneal hysteresis (CH). Three measurements were taken from each eye and the measurement with the best waveform score was used for analysis. Only measurements with a waveform score > 5 were considered for inclusion. All IOP measurements were taken at 9 am.

### Imaging and image analysis

**Figure 1:**
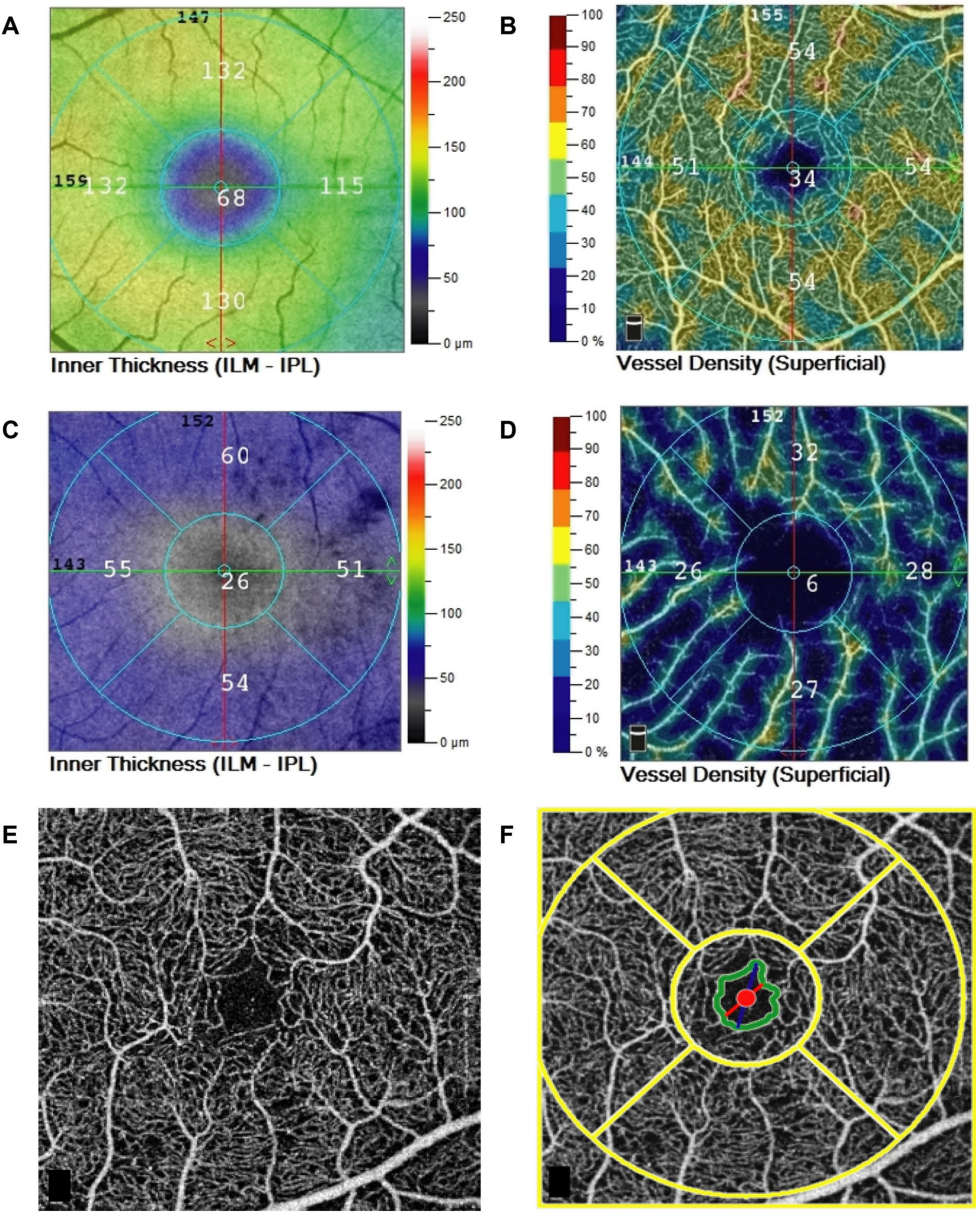
Example of OCT and OCTA images obtained from a healthy (**A**,**B**) and glaucomatous patient (**C**,**D**) showing inner macular thickness (IMT) (**A**,**C**) and superficial pfVD (**B**,**D**). En face macular OCTA images are shown (**E**,**F**), with (**F**) showing an OCTA image with the foveal avascular zone (FAZ) outlined (green ring).

OCT and OCTA imaging were performed using the RTVue XR Avanti (Optovue, Inc., Fremont, CA, U.S.A.). The RTVue uses an 840 nm near-infrared light source with a 50 nm bandwidth providing 70,000 A-scans per second. A three-dimensional optic nerve head scan was performed to obtain RNFL thickness measurements within a 4 mm diameter circle centered on the optic disc. Each optic nerve head scan, which consisted of 12 radial lines and 6 concentric rings, was used to create a RNFL thickness map. RNFL measurement were taken from a 920 point 3.5 mm diameter sampling circle derived from the RNFL thickness map, with the sampling circle centered on the optic disc. Macula OCT scans were also obtained using a 3 × 3 mm volumetric scan from which average macular ganglion cell complex (mGCC) thickness and inner macular thickness (IMT) (defined as the distance between the ILM and IPL) (Fig. 1) could be determined using built-in segmentation software. mGCC thickness was defined as the thickness of the ganglion cell layer, inner plexiform layer and RNFL within the 3 × 3 mm macular cube scan. All OCT scans were reviewed at the time of imaging for artefact and segmentation errors, and if of insufficient quality were repeated. Scans were required to have a signal strength index ≥ 40 with no segmentation failure of artefacts.

OCTA images were obtained at the same sitting and performed using a 3 × 3 mm macula scan with the split spectrum amplitude-decorrelation angiography (SSADA) algorithm used to provide non-invasive visualization of the retinal vascular network. In-built software automatically calculates pfVD as a percentage of measured area occupied by flowing blood vessels defined as pixels with decorrelation values above a threshold. For this study pfVD in the superficial retinal capillary plexus was analyzed from the 3 × 3 mm macula scan centered on the fovea. The superficial capillary plexus was defined as vessels identified from the inner limiting membrane (ILM) to the posterior margin of the inner plexiform layer (IPL). The analysis focused on pfVD, measured in an annular region of the 3 × 3 mm macula scan with an inner diameter of 1 mm and outer diameter of 3 mm centered on the fovea (Fig. 1)^27^. The quality of OCTA scans was assessed and those with quality scores (calculated automatically by the device software) of ≤ 4, motion artefact, decentration or poor clarity were repeated. If the repeat scan was also of poor quality the eye was excluded from the analysis.

Superficial capillary plexus OCTA images were also analyzed with VAMPIRE (Universities of Edinburgh and Dundee, UK)^23^. Retinal vessels were segmented by denoting each pixel as vessel or non-vessel to produce segmented images^28^. Resulting images were skeletonized using interactive deletion of pixels using MATLAB’s *bwmorph* algorithm^29^. Fractal dimensions can be measured either using a monofractal or multifractal approach^30^. In this study, we analyzed the images using the multifractal technique which is considered superior at characterizing the retinal vascular tree in fundus imaging and which calculates multiple fractal dimensions from multiple randomly chosen points within the skeletonized retinal vessels. One dimension from the spectrum, D0, was selected as the most appropriate measure of fractal properties of the retinal vessels based on previous work involving fundus imaging^30^.

### Statistical analysis

Descriptive statistics included mean and standard deviation (SD) for normally distributed variables, with normality tested by inspection of histograms and using Shapiro–Wilk test. Student t-test was conducted to examine differences in ocular and OCT parameters between better eyes and worse eyes, with better eye defined as the eye with better SAP MD. The relationship between OCT and OCTA parameters and potential confounders including age and OCT scan quality score was also explored using univariate regression analyses. Parameters that are found to be significant on univariate regression analyses were further investigated using multivariable regression analyses to determine whether relationships remained after accounting for the effect of age and scan quality.

Regression analyses assume residuals are independent, however, there is likely to be correlation between eyes of an individual. To avoid loss of data from excluding eyes, analyses were conducted comparing worse and better eyes and regression analyses was performed with standard errors adjusted to take into account observations within individuals being non-independent. Standard errors were calculated based on aggregate scores from individual patients using the STATA ‘cluster’ option^31^. All statistical analyses were performed in STATA (version 15.1; StataCorp LP, College Station, TX). The alpha level (type I error) was set at 0.05.

## Results

The study included 70 eyes of 35 patients, however 14 eyes were excluded due to poor quality OCTA images, leaving 57 eyes of 31 patients. Demographic and clinical characteristics of participants are summarized in Table 1. Participants had an average age of 66.2 ± 7.9 years, 16 of 32 (50%) were female and 14 of 32 (43.8%) had a history of glaucoma in a first degree relative.

**Table 1:** Demographic and clinical characteristics of patients with normal tension glaucoma included in the study.

Age (years)	66.2 ± 7.9		
Sex (female)	16 (50%)		
Family history (%)	14 (43.8%)		
Systemic hypertension (%)	17 (29.8%)		

	Worse eye (n = 31)	Better eye (n = 26)	*P* value
CCT (μm)	544.8 ± 33.7 (IQ range 523 to 565)	542.8 ± 35.5 (IQ range 516 to 570)	0.841
CH (mmHg)	9.8 ± 1.1 (IQ range 8.7 to 10.7)	9.8 ± 0.9 (IQ range 9.2 to 10.5)	0.930
IOPg—9am (mmHg)	17.5 ± 4.9 (IQ range 14.4 to 20.5)	17.1 ± 4.5 (IQ range 13.9 to 19.5)	0.808
IOPcc—9am (mmHg)	18.4 ± 4.3 (IQ range 16.1 to 21.0)	18.1 ± 4.0 (IQ range 15.8 to 19.8)	0.779
MD (dB)	− 6.5 ± 5.0 (IQ range − 8.7 to − 2.9)	− 1.4 ± − 1.8 (IQ range − 2.9 to − 0.6)	** < 0.001**
mGCC (μm)	78.7 ± 8.1 (IQ range 73.0 to 84.0)	82.0 ± 8.0 (IQ range 77.0 to 87.0)	0.134
cpRNFL (μm)	75.8 ± 14.1 (IQ range 70.0 to 80.0)	77.7 ± 12.0 (IQ range 69.0 to 85.0)	0.596
IMT (μm)	88.5 ± 12.2 (IQ range 77.8 to 95.4)	84.4 ± 12.9 (IQ range 74.4 to 93.2)	0.231
pfVD (%)	38.4 ± 4.9 (IQ range 35.6 to 42.6)	41.3 ± 2.9 (IQ range 39.8 to 42.8)	**0.012**
MFSD	1.82 ± 0.02 (IQ range 1.81 to 1.82)	1.81 ± 0.02 (IQ range 1.80 to 1.82)	0.636
FAZ perimeter (mm)	2.26 ± 0.61 (IQ range 1.70 to 2.27)	2.27 ± 0.52 (IQ range 1.76 to 2.70)	0.941

Values with a *P*-value < 0.05 are highlighted in bold.

MD in the worse and better eyes was − 6.5 ± 5.0 and − 1.4 ± − 1.8 dB respectively (*P* < 0.001), with cpRNFL thicknesses of 75.8 ± 14.1 and 77.7 ± 12.0 μm in worse and better eyes respectively (*P* = 0.596). pfVD was 38.4 ± 4.9 and 41.3 ± 2.9% in worse and better eyes (*P* = 0.012). There was no significant difference in FAZ perimeter between worse and better eyes (*P* = 0.941).

Regression analyses found a significant relationship between thinner mGCC (R^2^ = 0.22), thinner cpRNFL (R^2^ = 0.12), lower pfVD (R^2^ = 0.14), and worse MD (Table 2). MD decreased by 0.26 dB (95% CI 0.12 to 0.40 dB, *P* < 0.001) for every 1 μm decrease in mGCC thickness, by 0.12 dB (95% CI 0.03 to 0.21 dB, *P* = 0.010) for every 1 μm decrease in cpRNFL and by 0.38 dB (95% CI 0.11 to 0.65 dB, *P* = 0.007) for every 1% decrease in pfVD.

**Table 2:** Results of univariable regression analyses examine the relationship between MD and other variables.

	Coefficient/odds ratio*	95% CI	*P* value
Age (years)	− 0.004	− 0.161 to 0.152	0.958
Gender* (female)	0.975	0.861 to 1.105	0.695
Hypertension* (yes)	0.951	0.839 to 1.079	0.439
CCT (μm)	− 0.101	− 0.046 to 0.026	0.577
CH (mmHg)	− 0.207	− 1.626 to 1.033	0.656
IOPg (mmHg)	0.103	− 0.178 to 0.384	0.465
IOPcc (mmHg)	0.127	− 0.188 to 0.443	0.420
mGCC (μm)	**0.261**	**0.123 to 0.399**	**< 0.001**
cpRNFL (μm)	**0.119**	**0.029 to 0.209**	**0.010**
IMT (μm)	0.094	− 0.002 to 0.190	0.056
pfVD (%)	**0.377**	**0.109 to 0.645**	**0.007**
MFSD	− 64.83	− 133.36 to 3.69	0.063
FAZ perimeter (mm)	0.566	− 1.700 to 2.831	0.619

Values with a *P*-value < 0.05 are highlighted in bold.

**Figure 2:**
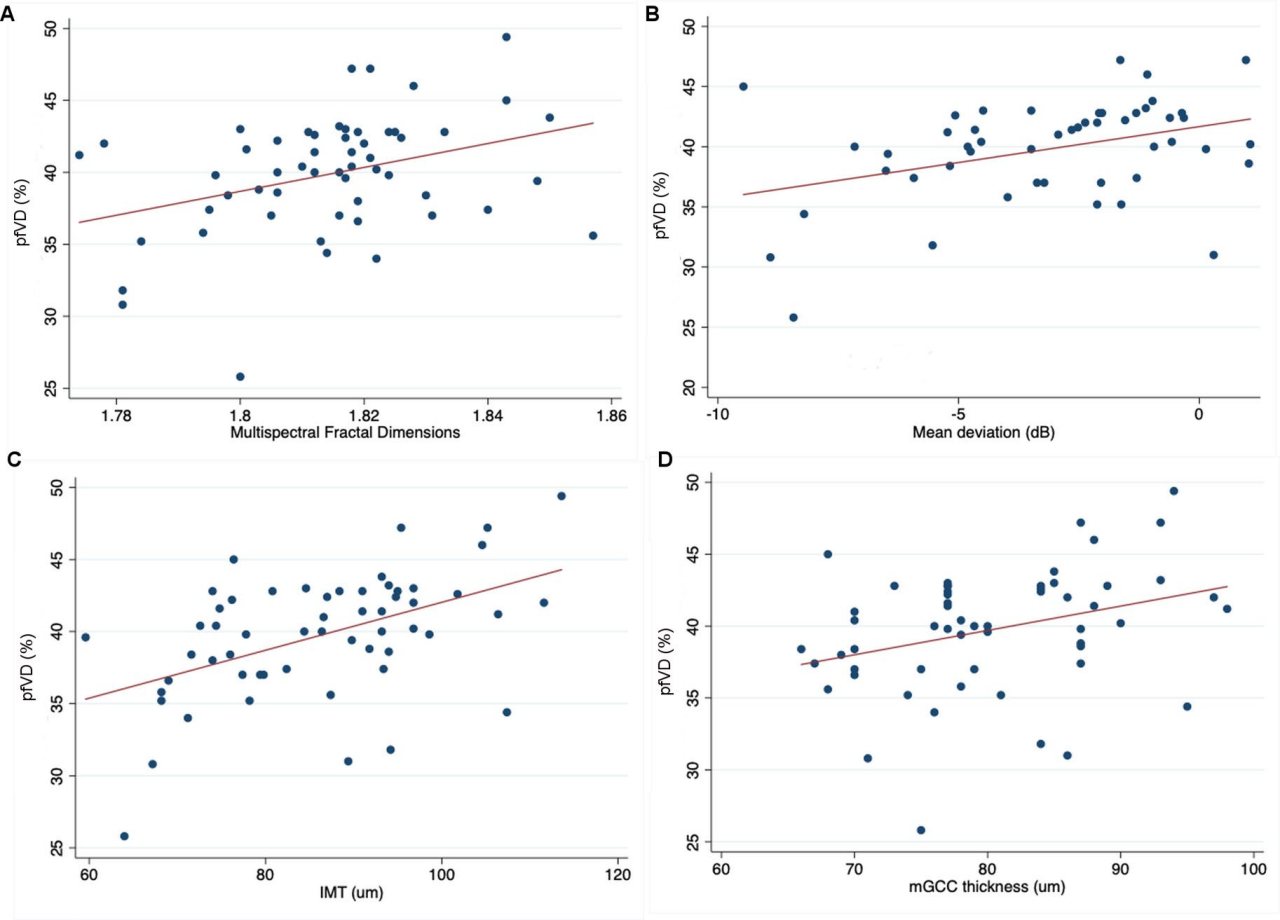
(**A**) Relationship between multispectral fractal dimensions and average pfVD (R^2^ = 0.124, *P* = 0.008). (**B**) Relationship between MD and average pfVD (%) (R^2^ = 0.136, *P* = 0.007). (**C**) Relationship between IMT and average pfVD (%) (R^2^ = 0.237, *P* < 0.001). (**D**) Relationship between IMT and average pfVD (%) (R^2^ = 0.104, *P* = 0.015).

There was also a significant relationship between lower pfVD and older age (R^2^ = 0.14), thinner mGCC (R^2^ = 0.10), thinner IMT (R^2^ = 0.24), lower MFSD (R^2^ = 0.11) and worse OCTA scan quality score (R^2^ = 0.32) (Table 3, Fig. 2). pfVD decreased by 0.17% (95% CI 0.03 to 0.21, *P* = 0.015) for every 1 μm decrease in mGCC, by 0.17% (95% CI 0.09 to 0.25, *P* < 0.001) for every 1 μm decrease in IMT, and by 82.9% (95% CI 19.81 to 146.01, *P* = 0.011) for every 1 μm decrease in MFSD. pfVD was also significantly higher with better OCTA quality score, and younger age, with a 0.21% (95% CI 0.35 to 0.07, *P* = 0.004) decrease in pfVD per year older.

**Table 3:** Results of univariable regression analyses examine the relationship between pfVD and other variables.

	Coefficient/odds ratio*	95% CI	*P* value
Age (years)	**− 0.21**	**− 0.35 to − 0.07**	**0.004**
Gender* (female)	0.960	0.846 to 1.089	0.525
Hypertension* (yes)	0.877	0.759 to 1.012	0.072
CCT (μm)	− 0.010	− 0.045 to 0.024	0.554
CH (mmHg)	0.527	− 0.529 to 1.582	0.321
MD (dB)	**0.359**	**0.104 to 0.615**	**0.007**
IOPg (mmHg)	− 0.118	− 0.355 to 0.118	0.320
IOPcc (mmHg)	− 0.166	− 0.431 to 0.100	0.213
mGCC (μm)	**0.170**	**0.034 to 0.205**	**0.015**
cpRNFL (μm)	0.063	− 0.027 to 0.153	0.165
IMT (μm)	**0.166**	**0.085 to 0.248**	**< 0.001**
MFSD	**82.91**	**19.81 to 146.01**	**0.011**
FAZ perimeter (mm)	− 2.02	− 4.10 to 0.06	0.057
OCTA quality	**2.046**	**1.227 to 2.865**	**< 0.001**

Values with a *P*-value < 0.05 are highlighted in bold.

**Figure 3:**
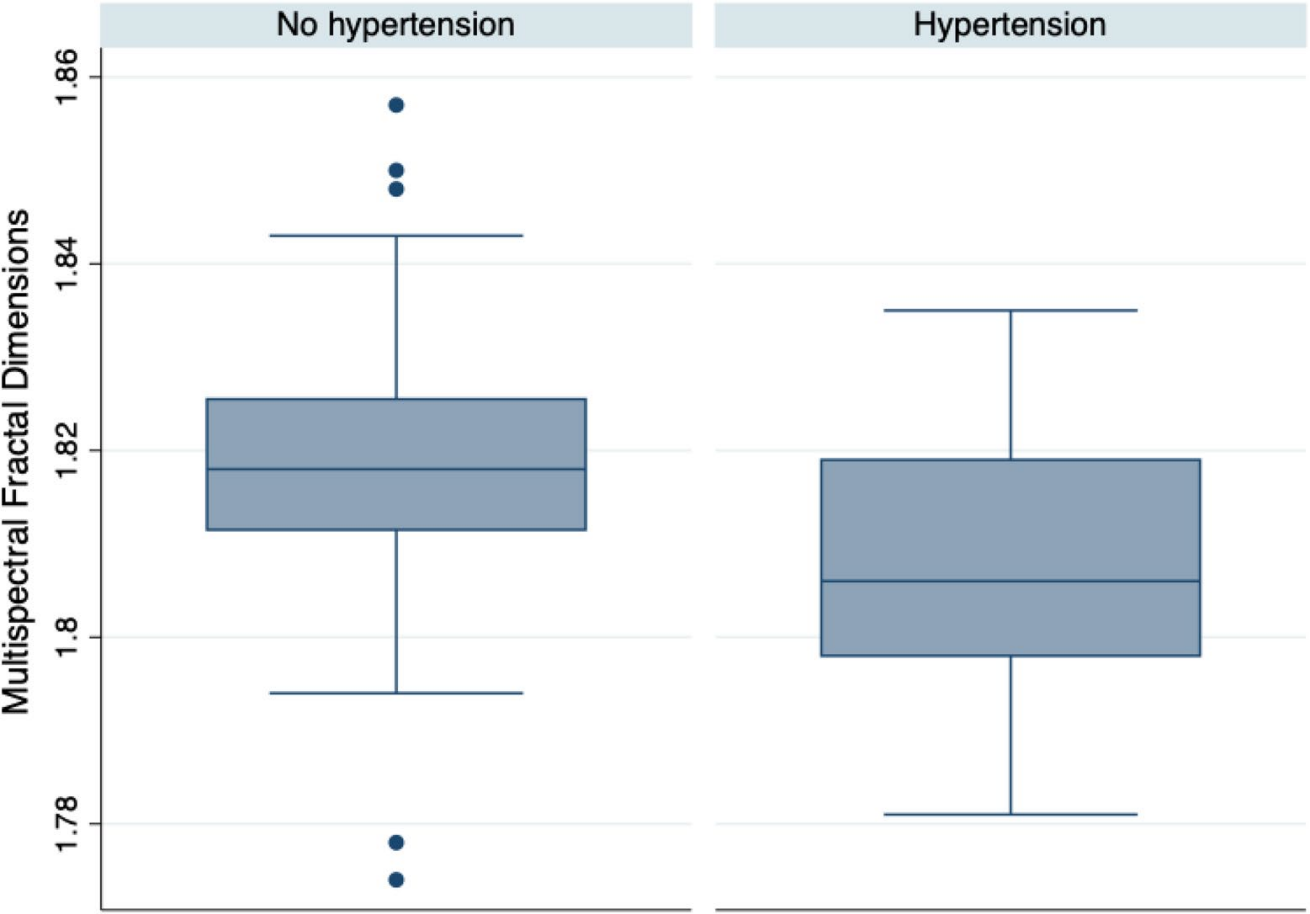
Box plot summarizing MSFD in glaucoma patients with and without systemic hypertension (*P* = 0.036).

Multispectral fractal dimensions ranged from 1.774 to 1.857. Lower multispectral fractal dimensions were found in eyes with lower pfVD, worse quality score, and in patients with systemic hypertension, but there were no other significant associations (Table 4). Mean MSFD was 1.82 ± 0.02 in non-hypertensives compared to 1.81 ± 0.02 in hypertensives (*P* = 0.036) (Fig. 3). A regression analysis examining the potential relationship between MD and multispectral fractal dimensions found a 0.1 increase in MSFD was associated with a 6.5 dB worse MD, however the 95% confidence interval crossed zero, at − 3.7 to 13.3 (*P* = 0.063), indicating non-significance.

**Table 4:** Results of univariable regression analyses examine the relationship between MSFD and other variables.

	Coefficient/odds ratio*	95% CI	*P* value
Age (years)	− 0.001	− 0.001 to 0.000	0.064
Gender* (female)	0.0001	5.31e−17 to 9.39e+09	0.638
Hypertension* (yes)	**5.62** × **10**^**−17**^	**8.68** × **10**^**−33**^ **to 0.364**	**0.044**
CCT (μm)	0.000	− 0.001 to 0.002	0.370
CH (mmHg)	0.000	− 0.005 to 0.004	0.960
MD (dB)	− 0.001	− 0.002 to 0.000	0.063
IOPg (mmHg)	0.00	− 0.00q to 0.001	0.838
IOPcc (mmHg)	0.00	− 0.001 to 0.001	0.871
mGCC (μm)	0.00	− 0.001 to 0.000	0.208
cpRNFL (μm)	0.00	0.00 to 0.00	0.808
pfVD (%)	**0.001**	**0.00 to 0.002**	**0.008**
IMT (μm)	0.00	0.00 to 0.001	0.420
FAZ perimeter (mm)	− 0.007	− 0.015 to 0.001	0.079
OCTA quality	**0.004**	**0.001 to 0.008**	**0.024**

Values with a *P*-value < 0.05 are highlighted in bold.

In multivariable analyses, accounting for age and quality score, lower pfVD on OCTA remained significantly associated with thinner IMT, worse MD and thinner mGCC, but not with MSFD (Table 5). The relationship between MSFD and hypertension remained after accounting for quality score in multivariable analysis (OR = 1.91 × 10^−21^, 95% CI 1.88 × 10^−39^ to 0.002, *P* = 0.024).

**Table 5:** Results of multivariable regression analyses examine the relationship between pfVD and IMT, MFSD, MD and mGCC, accounting for age, quality score and hypertension.

	Coefficient	95% CI	*P* value
**IMT (μm**)	0.15	0.08 to 0.21	< 0.001
Age (years)	− 0.09	− 0.20 to 0.02	0.104
Quality score	1.74	1.02 to 2.46	< 0.001
**MSFD**	29.63	− 30.33 to 89.59	0.217
Age (years)	− 0.06	− 0.22 to 0.11	0.495
Quality score	1.77	0.86 to 2.67	< 0.001
Hypertension (yes)	− 1.20	− 3.97 to 1.58	0.391
**MD (dB**)	0.38	0.18 to 0.58	< 0.001
Age (years)	− 0.14	− 0.26 to − 0.03	0.017
Quality score	1.59	0.82 to 2.37	< 0.001
**mGCC (μm**)	0.19	0.09 to 0.30	< 0.001
Age (years)	− 0.12	− 0.23 to − 0.00	0.045
Quality score	1.90	1.14 to 2.66	< 0.001

## Discussion

This study examined the relationship between established functional and structural markers of glaucoma and measures of retinal vasculature integrity in patients with NTG washed out of glaucoma medication. At present, OCTA devices perform only limited automated analyses, largely restricted to quantification of pfVD. We explored whether other characteristics of the superficial retinal capillary network might provide useful information in the evaluation of glaucoma, in particular, measurement of the complexity of capillary branching, measured as MSFD. MSFD is an interesting area for study given the changes in MSFD noted in patients with conditions such as hypertension and lacunar stroke and the role of ocular blood flow in the pathogenesis of glaucoma^23,24^.

In agreement with previous OCTA studies, we observed a significant relationship between worse MD and lower pfVD, which in this study was measured in the perifoveal superficial capillary network^9,12,32–37^. As expected, there was also a significant relationship between worse MD and thinner mGCC and cpRNFL (Table 2). There was a 0.377 dB (95% CI 0.109 to 0.645 dB, *P* = 0.007) decrease in MD for every 1% decrease in pfVD. In addition, pfVD was significantly lower in the worse eyes of glaucoma patients (*P* = 0.012), when worse eye was determined according to MD. pfVD was lower in eyes with thinner mGCC and IMT, and there was a relationship between lower pfVD and lower MSFD in univariable analysis. pfVD also decreased with age, at a rate of 0.21% (95% CI 0.07 to 0.35%) per year, however, even after accounting for age, and quality of the OCTA scan, the relationship between lower pfVD and thinner IMT, thinner mGCC and worse MD remained. In contrast, accounting for these confounders, the relationship between pfVD and MSFD was no longer significant.

MSFD was not significantly associated with MD, however, the 95% CI for the regression coefficient only just crossed zero (− 133.36 to 3.69, *P* = 0.063). The study was limited by a relatively small sample size and a lack of patients with severe glaucoma. It is therefore possible that the analysis was underpowered to detect an association between MSFD and MD and given the known association between MSFD and systemic vascular diseases, further study of MSFD in glaucoma is warranted. In addition, considering the relative lack of test points in the central visual field using the 24-2 SAP test pattern, it may also be useful to examine the relationship between MFSD and the results from 10-2 visual fields, however, this comparison may also find poor correlation, given the lack of relationship between MFSD and mGCC and IMT (Table 4).

The other novel vascular parameter investigated was FAZ perimeter, however this did not seem to be a useful marker of glaucoma in this particular cohort. There was no evidence of a significant difference in FAZ perimeter between better and worse eyes, and no relationship between MD and FAZ. Though the relationship between FAZ and pfVD was not statistically significant (*P* = 0.057, Table 3), again the 95% CI interval only just crossed zero (coefficient − 2.02, 95% CI − 4.10 to 0.06), and it may be worth examining this potential relationship in a larger cohort.

Unfortunately, 14 eyes were excluded from the analysis due to poor quality OCTA scans, and even in those remaining, OCTA quality score significantly affected measurements. For this reason, it was important to include quality score in the multivariable analyses examining the relationship between measurements obtained from OCTA and other parameters. Table 4 shows that even after accounting for quality score, pfVD remained associated with MD, mGCC thickness, and IMT. Interestingly, our sample included 17 patients (29.8%) on treatment for systemic hypertension. Although patients with hypertension had significantly lower MSFD (*P* = 0.036), hypertension was not associated with MD or pfVD on multivariate analyses. However, it does emphasize the potential value of measures of vascular branching complexity as a biomarker of systemic hypertension^23,24^.

We recognize that this study has several limitations and have already emphasized the small sample size and lack of patients with advanced glaucoma. Due to the glaucoma medication washout required for participation in this study, it was not deemed safe to include patients with advanced disease. Patients were asked to stop glaucoma medications to obtain a baseline IOP and due to the potential effects of IOP lowering medication on OCTA measurements, however, it would be useful to repeat the study without stopping medications to enable safe recruitment of more patients with advanced disease. The study was also limited by a cross-sectional design prohibiting analysis of the temporal relationship between changes to pfVD, MSFD, mGCC, cpRNFL and visual fields. In addition, we only examined average thickness and pfVD measurements and only examined one summary index of visual field loss. It is possible that examination of localized changes may reveal other associations, however, the small sample size and diversity of visual field defects did not make this feasible. A further limitation was that the diagnosis of systemic hypertension was based on history and patients were using a range of anti-hypertensive medications at the time of imaging.

In conclusion, this study provides further evidence of changes to retinal vasculature in glaucoma. Perifoveal vascular density was significantly lower in worse affected compared to fellow eyes and there was a significant relationship between pfVFD and MD. However, as pfVD also declines with age and is affected by quality of the OCTA image, it is important to take into account these potential confounders when interpreting pfVD data. Additional measures of integrity of retinal vasculature including MSFD and FAZ size were not related to markers of glaucoma severity and there was no significant difference in these parameters between better and worse eyes, however, MSFD was significantly reduced in NTG patients with systemic hypertension. There is growing evidence of potential value of MSFD as a biomarker for cardiovascular and neurodegenerative disease, which warrants further study in glaucoma.
